# Advanced Brain‐on‐a‐Chip for Wetware Computing: A Review

**DOI:** 10.1002/advs.202508120

**Published:** 2025-07-23

**Authors:** Shangchen Li, Yaoyao Liu, Sihan Hua, Yu Wang, Shutong Sun, Longhui Jiang, Chengji Lu, Juntao Liu, Huaizhang Shi, Pei Wu, Xinxia Cai, Jinping Luo

**Affiliations:** ^1^ State Key Laboratory of Transducer Technology Aerospace Information Research Institute Chinese Academy of Sciences Beijing 100190 China; ^2^ School of Electronic Electrical and Communication Engineering University of Chinese Academy of Sciences Beijing 100049 China; ^3^ Department of Neurosurgery The First Affiliated Hospital of Harbin Medical University Heilongjiang 150001 China

**Keywords:** MEA, MEMS, brain‐on‐a‐chip, microfluidics, wetware compute

## Abstract

In pursuit of low‐power consumption and surpassing computational limitations of silicon‐based chips, people are beginning to seek more efficient computing devices, such as Wetware Computing. The cutting‐edge approach uses living biological tissues, specifically neuronal networks, to perform computational tasks. This computing method, which is a mixture of hardware, software, and biology, is an emerging computing method that has received a lot of attention in recent years. As an important branch of organ‐on‐a‐chip, brain‐on‐a‐chip, which combines Micro‐Electro‐Mechanical System technology, electronic technology, and tissue engineering, can provide a powerful research platform for Wetware Computing. In this paper, the brain‐on‐a‐chip for Wetware Computing is reviewed. This paper summarizes the methods for establishing a brain‐on‐a‐chip for Wetware Computing, including the brain organoids cultured in vitro, microelectrode arrays, electrophysiology interfaces, and microfluidic platforms that make up the brain‐on‐a‐chip. In addition, the data processing methods of brain‐on‐a‐chip are reviewed, including encoding and decoding methods. In this paper, the focus is also on the application and the prospect of brain‐on‐a‐chip in Wetware Computing.

## Graphical abstract

Exploring Low‐Power, Beyond‐Silicon‐Computing Bio‐Computing: Brain‐on‐a‐Chip Technology. This article reviews the applications of Brain‐on‐a‐Chip in Wetware Computing, including in vitro‐cultured brain organoids, microelectrode arrays, electrophysiological interfaces, and microfluidic platforms, as well as data processing methods. It also looks forward to the future of Brain‐on‐a‐Chip in Wetware Computing, discussing both its potential and the challenges it faces.

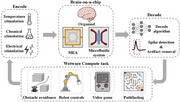

## 1 Introduction

In recent years, as deep learning and Large Language Model technologies have advanced significantly, there has been a substantial growth in computational requirements, model parameters, and energy usage during training, leading to escalating costs for developing cutting‐edge AI models.^[^ ^1^ ^]^ OpenAI's GPT‐4 demands an estimated $78 million in training expenditures, while Google's Gemini Ultra model is $191 million.^[^ ^2^ ^]^ The growth of AI technology has also increased the performance requirements for the carrier of its operation, silicon‐based chips. However, because of quantum interference, thermal fluctuations, and other influences, the manufacturing process of transistors is approaching the physical limit, and the power consumption problem is becoming more and more significant.^[^ ^3^ ^]^ The energy consumption, operating efficiency, computational load, and learning ability of traditional silicon‐based chips have gradually become bottlenecks.

To achieve stronger computing capabilities, people have carried out research in the direction of biological computing and biochips, which is also called “Wetware Computing”.^[^ ^4^ ^]^ Wetware Computing is an emerging field of computing that combines hardware, software, and biology. Its core is using biological tissues and cells to perform computational tasks. Wetware Computing has unparalleled potential in terms of energy consumption, parallel processing capabilities, and self‐learning capabilities,^[^ ^5^ ^]^ which enables Wetware Computing significant cost‐saving advantages compared to traditional silicon‐based AI training methods.

**Figure 1:**
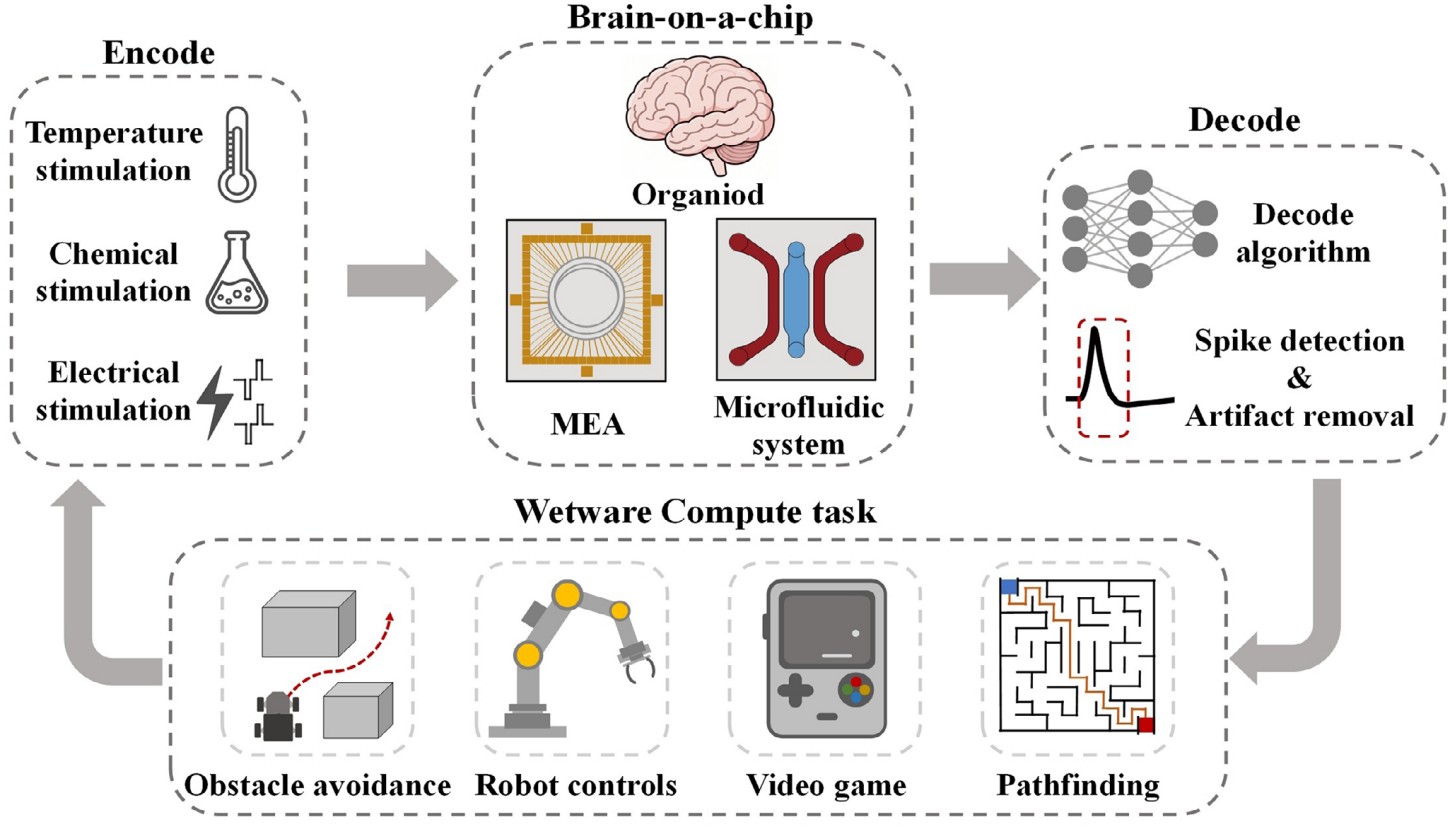
Brain‐on‐a‐Chip for Wetware Computing tasks. The Brain‐on‐a‐Chip system utilizes organoids as its core decision‐making unit, supported by a Micro‐Electrode Array (MEA) as the front‐end receptor and a microfluidic system for nutrient delivery and complex brain model construction. Information encoding in wetware computing tasks is achieved through various stimulation methods, including thermal, chemical, and electrical stimuli. To interpret the system's output, spike detection and artifact removal technologies are employed alongside advanced decoding algorithms. This platform is capable of performing diverse wetware computing tasks, such as obstacle avoidance, robot control, video game, and pathfinding.

The use of neural networks cultured in vitro and brain organoids can respond to external stimuli through electrophysiological activities, to realize the functions of data calculation and decision‐making, which is a commonly used method for constructing Wetware Computing equipment. Numerous studies have shown that through certain training, neural networks cultured in vitro and brain organoids can realize specified tasks, such as controlling the robotic arm,^[^ ^6^ ^]^ obstacle avoidance,^[^ ^7^ ^]^ speech recognition,^[^ ^8^ ^]^ playing games in the virtual world^[^ ^9^ ^]^ and others (**Figure** **1**). Biochips have shown better performance than silicon‐based chips in terms of power consumption, learning ability, and training speed. Compared with planar 2D cultured neural networks, brain‐on‐a‐chip has advantages in terms of neuronal number, neuronal type, computing performance, and working life, which makes it have broad development prospects in the field of Wetware Computing.^[^ ^10^, ^11^ ^]^ The purpose of this paper is to review the architectural design of brain‐on‐a‐chip and the data processing algorithms required by brain‐on‐a‐chip for Wetware Computing tasks, and summarize the application performance of brain‐on‐a‐chip in Wetware Computing, and look forward to the prospect of it.

## 2 Architectural Design of Brain‐on‐a‐Chip

Constructing a complete brain‐on‐a‐chip is a prerequisite for its application in Wetware Computing. The brain‐on‐a‐chip system comprises three fundamental elements: the brain model, microelectrode arrays and interface circuits, and the microfluidic platform, each of which plays a crucial role.

### 2.1 In Vitro Neuronal Cultures for Brain Organoid Models

The main computing function of brain‐on‐a‐chip is realized by neurons or brain organoids on the chip, which is the main body of computing and decision‐making for Wetware Computing tasks. According to the structure of cultured cells, it can be divided into: the cultivation of 2D neural networks and the cultivation of 3D brain organoids.^[^ ^12^ ^]^

#### 2.1.1 Biological Neural Network Model Developed through 2D Cultivation

**Figure 2:**
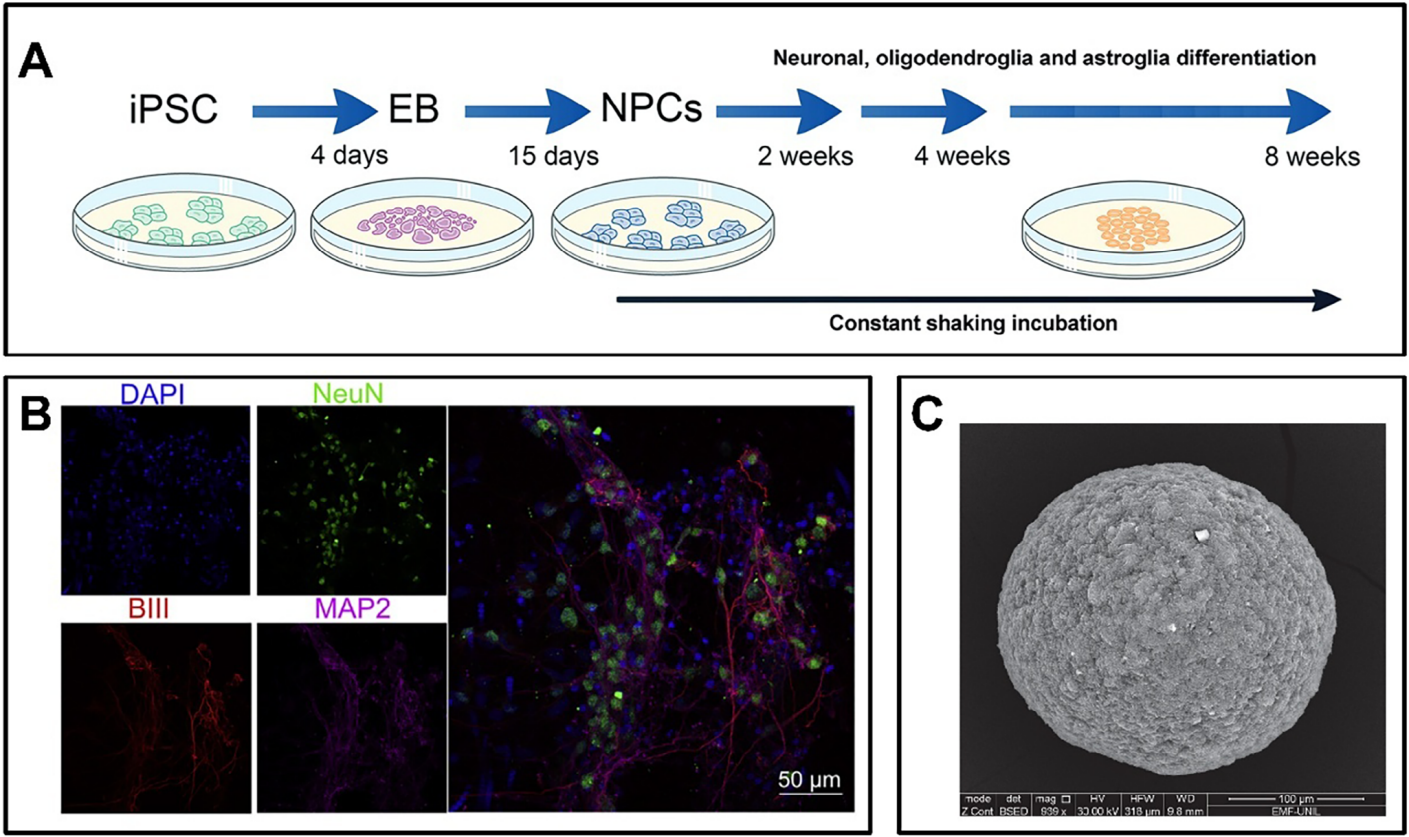
In vitro culture of brain organoid models. A) Common differentiation protocol from iPSCs.^[^ ^33^ ^]^ B) Cortical cells differentiated from hiPSCs.^[^ ^9^ ^]^ DAPI (blue) stains all cells, NeuN (green) marks neurons, BIII (red) labels axons, and MAP2 (purple) labels dendrites. C) Scanning electron microscopy image of a brain organoid (scale bar = 100 µm), which shows that it is a compact spheroid.^[^ ^34^ ^]^ (A) Reproduced under terms of the CC‐BY license.^[^ ^33^ ^]^ Copyright 2016, published by Springer Spektrum. (B) Reproduced under terms of the CC‐BY license.^[^ ^9^ ^]^ Copyright 2022, published by Elsevier. (C) Reproduced under terms of the CC‐BY license.^[^ ^34^ ^]^ Copyright 2024, published by Frontiers.

The cultivation of 2D neural networks often uses neural stem cells (NSCs),^[^ ^13^ ^]^ embryonic stem cells (ESCs),^[^ ^14^ ^]^ induced pluripotent stem cells (iPSCs)^[^ ^15^, ^16^ ^]^ or primary neurons isolated from neural tissue^[^ ^17^ ^]^ (**Figure** **2**A). Neural cells exhibit significant dependence on the extracellular matrix (ECM), so we need to coat the dish before cultivation. The commonly used coating materials include poly‐L‐lysine (PLL), poly‐D‐lysine (PDL), laminin, etc. Once coated, neurons can be seeded, and media should be added to the dish with tight control of temperature, humidity, and oxygen levels during the cultivation process. If stem cells are utilized, specific inducers are needed to promote differentiation; these may include retinoic acid, Noggin, and Sonic Hedgehog (Shh). Once those are done, the neurons will gradually extend their axons and dendrites to form synaptic connections with each other. Typically, 2–3 days after seeding, the cells will start to form neurites, and after 1 and 2 weeks, initial neuronal networks will be established. It is crucial to replace a portion of the medium (30%–50%) every 2–3 days to sustain nutrient supply and eliminate waste products (Figure 2B).

2D neural cell networks can realize the functions of biological neural networks and can respond to external stimuli effectively, but the computational performance of 2D neural networks is relatively poor.^[^ ^18^ ^]^ This is because 2D‐cultured nerve cells are often flattened and have relatively simple connections between cells, which do not fully mimic the complex neuronal network inside the brain.^[^ ^19^ ^]^ Furthermore, the signal transmission between 2D cultured nerve cells is constrained by the culture conditions, leading to low transmission efficiency and an inability to fully simulate the complex signal processing that occurs in the brain. Those limit the performance of 2D neural networks in Wetware Computing, making it unable to handle complex computing tasks or build large‐scale neural network models.

#### 2.1.2 Brain Organoids Developed Through 3D Cultivation

Organ‐on‐a‐Chip is a microfluidic cell culture device manufactured by the microchip manufacturing method^[^ ^20^ ^]^ to simulate and reconstruct the physiological functions of human organs,^[^ ^21^, ^22^ ^]^ including the brain. Brain‐on‐a‐chip is the product of combining brain organoids with organ‐on‐a‐chip technology. 3D brain organoids possess better computing and storage power than 2D neural networks due to their complex structure (Figure 2C). However, the difficulty and cost of cultivating 3D brain organoids are much higher than those of 2D neural networks. At present, most of the in vitro chips used for Wetware Computing utilize nerve cells cultured in 2D.

Compared to 3D brain organoids, 2D nerve cells have many shortcomings. First, Brain organoids exhibit tissue structure and function similar to those of the human brain.^[^ ^23^ ^]^ For instance, brain organoids can contain myelinated axons,^[^ ^24^ ^]^ display better physiological self‐generated activity,^[^ ^25^ ^]^ and demonstrate complex oscillatory behavior.^[^ ^26^ ^]^ As a result, the information processing capacity and speed of brain organoids exceed those of 2D nerve cells. Additionally, the cell density in 3D brain organoids is significantly higher than that of monolayer‐cultured nerve cells. This increased cell density enables brain organoids to achieve greater information storage capacity.^[^ ^5^ ^]^ Besides, 3D brain organoids contain more cell types,^[^ ^27^ ^]^ and non‐neuronal cells (e.g., oligodendrocytes, microglia, and astrocytes) make significant contributions to neural learning mechanisms.^[^ ^28^ ^]^ Experiments have proven that 3D brain organoids have the characteristics of nonlinear dynamics and spatial information processing required for Wetware Computing and have a high accuracy in a variety of Wetware Computing tasks.^[^ ^8^ ^]^

Stem cell technology can culture stem cells with long‐term multiplication and self‐renewal capabilities,^[^ ^29^ ^]^ so it plays an indispensable role in the construction of 3D brain organoid models cultured in vitro. Current brain organoid culture systems predominantly utilize two stem cell sources: embryonic stem cells (ESCs) and induced pluripotent stem cells (iPSCs). The operation of the two stem cells to generate brain organoids is similar and typically includes the cultivation and expansion of stem cells, the generation of embryoid bodies (EBs), the neural induction of embryoid bodies, and the reaggregation of SFEBq or Matrigel media,^[^ ^8^, ^30^ ^]^ long‐term culture, and tissue differentiation.^[^ ^31^, ^32^ ^]^

### 2.2 Micro‐Electrode Array and Electrophysiological Interface for Brain‐on‐a‐Chip

Currently, most applications utilizing biochips for Wetware Computing rely on electrophysiological stimulation and detection to encode and decode information. Micro‐Electrode Arrays, commonly used as front‐end receptors, provide channels that offer a high signal‐to‐noise ratio and excellent stability for both the output of electrophysiological signals and the input of electrical stimulation. The electrophysiological interface, which typically includes an electrical stimulator and signal‐acquiring device, can achieve high‐sensitivity, high‐tunability electrical stimulation signal input and high‐resolution, wide‐range electrophysiological signal readout, respectively.

#### 2.2.1 The Front‐End Receptor–The Micro‐Electrode Array

The Micro‐Electrode Array (MEA) is a widely used platform in biochips. It is capable of recording and stimulating neuronal electrical activity, making it a crucial tool in neuroscience research. Relying on its tiny electrodes, MEA can monitor cellular action potentials, synaptic transmission, and network activity.^[^ ^35^, ^36^ ^]^

Common MEAs include MEMS‐MEAs fabricated by Micro‐Electro‐Mechanical System (MEMS) technology and CMOS‐MEAs fabricated by Complementary Metal‐Oxide‐Semiconductor (CMOS) processes. MEMS‐MEA can accommodate several electrode sites, ranging from tens to hundreds.^[^ ^35^, ^37^, ^38^ ^]^ In contrast, CMOS‐MEA can support thousands or even tens of thousands of electrode sites due to the capabilities of the CMOS process.^[^ ^39^, ^40^ ^]^ CMOS‐MEA offers ultra‐high spatiotemporal resolution and facilitates noise reduction, signal stabilization, and signal amplification, thereby enhancing the quality of the input electrical signals.^[^ ^41^, ^42^ ^]^ MEMS‐MEA has its advantages, such as lower manufacturing cost and simpler manufacturing process, while CMOS‐MEA has the advantages of achieving high electrode density and high signal quality.^[^ ^39^ ^]^ In addition, MEMS technology offers enhanced flexibility in electrode design, allowing for the creation of 3D electrode arrays. This capability is particularly beneficial for 3D brain organoids. Currently, MEMS‐MEA is predominantly utilized as the front‐end receptor within brain‐on‐a‐chip systems.

**Figure 3:**
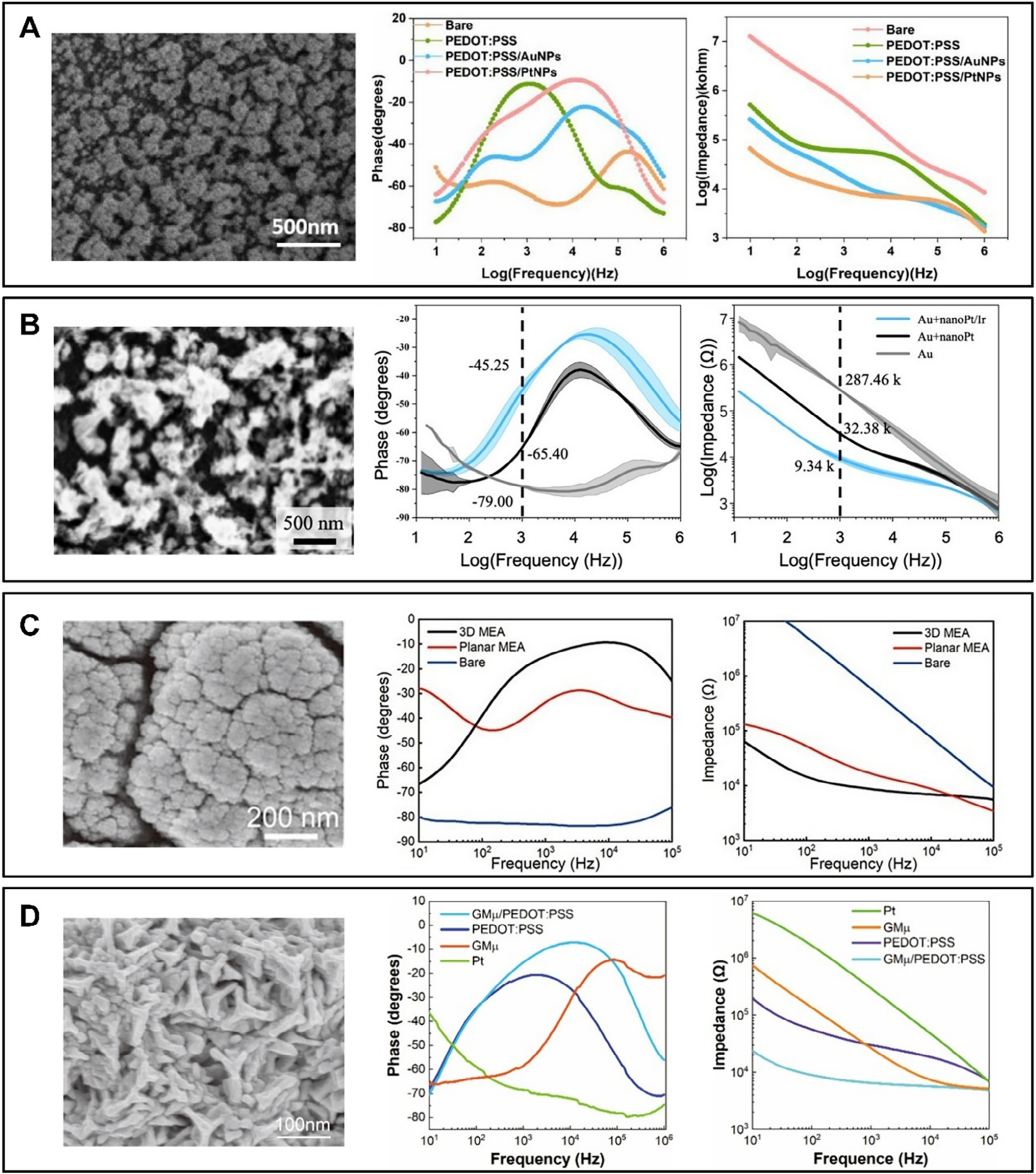
Morphological and electrical characteristics of electrodes with different modification methods. A) SEM image of PEDOT:PSS/PtNPs and impedance and phase properties of bare Pt electrodes, PEDOT:PSS, PEDOT:PSS/AuNPs, and PEDOT:PSS/PtNPs modified Pt electrodes from 10 Hz to 1 MHz.^[^ ^38^ ^]^ B) SEM image of nanoPt/Ir and impedance and phase properties of bare Au electrodes, nanoPt and nanoPt/Ir modified Au electrodes from 10 Hz to 1 MHz.^[^ ^50^ ^]^ C) SEM image of 3D MEA and impedance and phase properties of bare electrodes, planar MEA, and 3D MEA modified by PtNPs from 10 Hz to 0.1 MHz.^[^ ^44^ ^]^ D) SEM image of 3D‐GMµE and impedance and phase properties of bare Pt electrodes, GMµEs, PEDOT:PSS, and GMµEs/PEDOT:PSS modified Pt electrodes from 10 Hz to 0.1 MHz.^[^ ^46^ ^]^ (A) Reproduced with permission.^[^ ^38^ ^]^ Copyright 2022, published by American Chemical Society. (B) Reproduced with permission.^[^ ^50^ ^]^ Copyright 2024, published by American Chemical Society. (C) Reproduced under terms of the CC‐BY license.^[^ ^44^ ^]^ Copyright 2023, published by Frontiers. (D) Reproduced under terms of the CC‐BY license.^[^ ^46^ ^]^ Copyright 2024, published by American Chemical Society.

The fabrication of MEMS‐MEA devices generally follows a multi‐stage sequence: substrate selection, substrate cleaning, spin coating photoresist, mask exposure, development, deposition of electrode patterns, deposition of insulation, and etching.^[^ ^43^ ^]^ Stimulation electrodes tend to be made in larger sizes for more efficient electrical stimulation signal transmission, while recording electrodes tend to be smaller in size for more spatially accurate detection.^[^ ^41^ ^]^ In addition to improving the recording and stimulation ability of MEA, a variety of nanomaterials are often used to modify the electrode, and those nanomaterials reduce the phase angle and impedance of the electrode.^[^ ^44^, ^45^, ^46^, ^47^, ^48^ ^]^ Commonly used modifiers include organic polymers like PEDOT:PSS (**Figure** **3**A), metal nanoparticles such as nanoPt/Ir (Figure 3B), and carbon‐based nanomaterials. The modified electrode surface forms a porous and rough structure, consequently enhancing both the recording fidelity and electrical stimulation efficacy of the electrode.^[^ ^38^, ^49^, ^50^ ^]^ Additionally, modifying the electrodes into a 3D structure can also significantly enhance their performance.^[^ ^44^, ^46^ ^]^ (Figure 3C,D) (It is important to clarify that the 3D structure mentioned here refers specifically to the configuration of the electrodes themselves, which is distinct from the 3D structure of the microelectrode arrays that will be discussed later.) Furthermore, the microscopic images reveal the surface morphology of the electrodes, showing the formation of a rough and porous structure due to the nanomaterial modifications, which are intentionally designed to enhance electrode performance by increasing the specific surface area.

Currently, many brain‐on‐a‐chip systems utilize 2D MEA as electrodes to control and adjust the biological neural networks grown on it.^[^ ^44^ ^]^ For example, the Neuroplatform,^[^ ^34^ ^]^ a mature brain‐on‐a‐chip research platform, incorporates four 2D MEAs. Each MEA can accommodate four organoids, and each organoid is equipped with eight electrodes for recording and stimulation. Moreover, higher neural network plasticity can be achieved by optimizing the structure of the 2D MEA, which in turn enhances the performance of the neural network during stimulation.^[^ ^51^ ^]^ However, since brain organoids are predominantly spherical, 3D MEA can provide better connectivity with these tissues. This improves the quality of the signal and sensitivity of detection. Compared with 2D MEA, 3D MEA can record more action potentials simultaneously under the same conditions, resulting in a higher signal‐to‐noise ratio and greater sensitivity of the recorded signals.^[^ ^47^ ^]^

**Figure 4:**
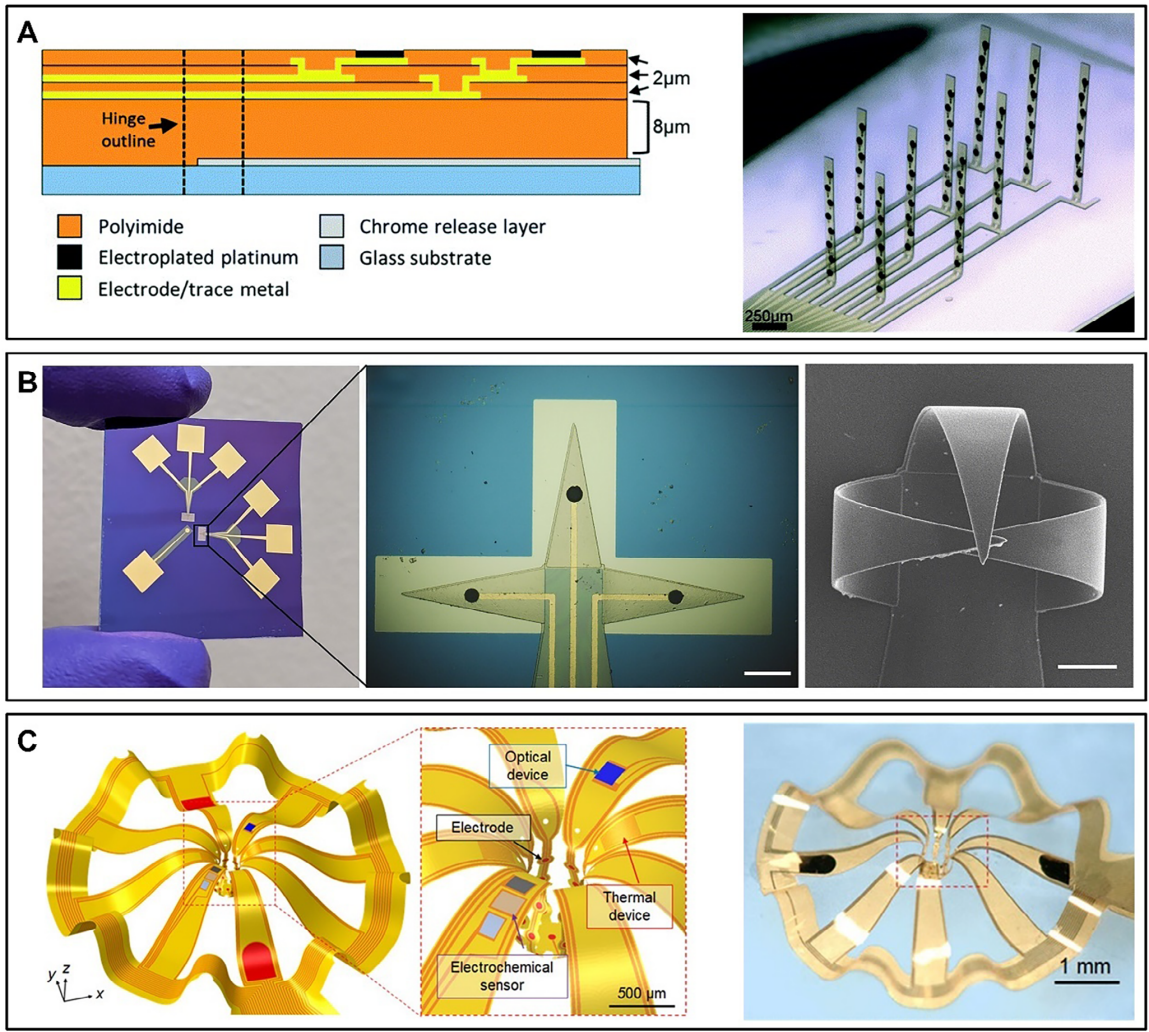
Different types of 3D microelectrode arrays for brain‐on‐a‐chip. A) Schematic of the 3D MEA with hinges for an upright probe, and a 3D MEA optical micrograph after initiation.^[^ ^55^ ^]^ B) Physical image of the 3D shell MEA, and a magnified optical image of the electrode of the shell in a flat state (scale bar = 200 µm), and scanning electron microscopy image (scale bar = 100 µm) after driving.^[^ ^47^ ^]^ C) The FEA result of the 3D MEA fabricated by “planar, lithographically fabricated multilayer stack” technology and a zoomed‐in view highlighting the functional components, including the 25 microelectrodes, as well as optical micrographs.^[^ ^56^ ^]^ (A)Reproduced under terms of the CC‐BY license.^[^ ^55^ ^]^ Copyright 2020, published by Royal Society of Chemistry. (B) Reproduced under terms of the CC‐BY license.^[^ ^47^ ^]^ Copyright 2022, published by the American Association for the Advancement of Science. (C) Reproduced under terms of the CC‐BY license.^[^ ^56^ ^]^ Copyright 2021, published by the American Association for the Advancement of Science.

Back in 2003, Shoji Takeuchi et al.^[^ ^52^ ^]^ developed a 3D structure by embedding ferromagnetic metals within the polyimide probe layer and utilized an industrial solenoid to position the probe upright. However, this approach requires ferromagnetic metals such as nickel, which are often cytotoxic and can affect the longevity and activity of brain organoids.^[^ ^53^ ^]^ Another way to prepare 3D MEA is to apply an external electrostatic field of 8 kV to a designed flexible microneedle array, using an electrostatic drive to fold the planar probe into a 3D probe in any direction.^[^ ^54^ ^]^ This approach solves the cytotoxicity of ferromagnetic metals, but using the externally energized device to perform the drive poses a potential hazard. The 3D structure of the MEA can also be made by pre‐designing the hinge area on the MEA and using a drive with a “buckling shank” and a “lifting shank” to bend the probe body away from the surface and then using a rigid structure to erect it^[^ ^55^ ^]^ (**Figure** **4**A). This method reduces the risk to brain organoids and greatly improves preparation efficiency, completing the 3D transformation of MEA in under 5 min. Qi Huang et al.^[^ ^47^ ^]^ utilized a negative photoresist polymer (SU8) with self‐folding properties to fabricate MEAs, and by irradiating it with ultraviolet light to fold SU8, the 3D transformation of MEAs was achieved. The folding angle can be adjusted by controlling the thickness of SU8 and the intensity of ultraviolet light (Figure 4B). Yoonseok Park et al.^[^ ^56^ ^]^ utilized “Planar, Lithographically Fabricated Multilayer Stack” technology^[^ ^57^ ^]^ to make MEA with a 3D structure(Figure 4C). This method requires pre‐strained elastomer substrates, which may limit integration with other modules or microfluidic devices. In addition, as a cutting‐edge technology, 3D printing of metal nanoparticles can also construct highly customizable, structurally robust, and high‐density microelectrode arrays.^[^ ^58^ ^]^

#### 2.2.2 The Electrophysiology Interface Realizes the Electrophysiological Interaction of Brain Organoids

The electrophysiology interface usually includes two parts, namely the electrical signal collector and the electrical signal stimulator, which correspond to the data acquisition and stimulation transmission of the brain organoids, respectively. Through the electrical signal collector, the electrophysiological activity of brain organoids can be detected, and their stimulation response can be obtained. Efficient stimulation of brain organoids can be achieved using an electrical signal stimulator, altering their electrophysiological activity.

Designing a high‐performance electrical signal collector is essential for accurately reading electrophysiological data from brain‐on‐a‐chip systems, allowing for the extraction of high‐quality signals from brain organoids.^[^ ^59^ ^]^ Typically, electrical signal acquisition systems include components such as low‐noise instrumentation amplifiers (LNIA), filters, analog‐to‐digital converters (ADC), etc. Action potentials are usually electrophysiological signals that need to be detected in the brain‐on‐a‐chip. In the resting state, the potential difference between the inside and outside of the neuronal membrane is −70 mV. When neuronal electrical stimulation occurs and reaches the threshold of −50 mV, a sharp wave discharge will be quickly generated on the neuronal membrane, that is, the action potential with an amplitude between 90–130 mV, also known as “Spike”.^[^ ^60^, ^61^ ^]^ Under normal circumstances, the frequency range of action potential of nerve cells is roughly 1Hz∼10 Hz, and the frequency of action potential emission can reach ≈300 Hz under external stimulation.^[^ ^62^ ^]^ Take *f_max_* as 300 Hz, and according to the Nyquist − Shannon sampling theorem,  the sampling rate *f_s_* only needs to be greater than 600 Hz. To completely satisfy the acquisition requirements for neuronal action potentials, the electrophysiological acquisition system usually adopts a sampling frequency of ≈25 kHz^[^ ^62^ ^]^ and bandpass filtering using a filter with a pass band of 1–3000 Hz.^[^ ^63^ ^]^ In addition, to achieve distortion‐free amplification, the input voltage range of the amplifier circuit should at least cover the range from the resting state potential to the action potential amplitude, that is, −70–130 mV. Universal acquisition boards are also commonly used by researchers to collect electrophysiological signals.^[^ ^7^, ^8^, ^9^, ^34^, ^64^ ^]^

Designing a reliable and stable stimulator is also a complex engineering challenge. Electrophysiological stimulators generally consist of waveform memory, digital‐to‐analog converters (DACs), amplifiers, and filters. By incorporating pre‐filtering within the electrical stimulator, it is possible to achieve energy savings of up to 51.5% in electrical stimulation.^[^ ^51^ ^]^ The Easypace pulse generator based on Arduino was designed as an electrical stimulation device for heart‐on‐a‐chip ^[^ ^65^ ^],^ and its DAC can generate two independent output signals, which are subsequently converted to biphasic pulses via an operational amplifier to realize the stimulation function. There are also several researchers who have utilized established commercial stimulators for electrophysiological stimulation.^[^ ^7^, ^8^, ^9^, ^34^, ^64^, ^66^ ^]^

### 2.3 Microfluidic Platform for Brain‐on‐a‐Chip

Most of the existing in vitro brain organoids still lack mature structural tissue, and there are limitations on tissue size. The lack of vascular perfusion limits nutrient supply to brain organoids, as diffusion becomes insufficient to meet rising metabolic requirements, inevitably leading to central necrosis during maturation.^[^ ^67^ ^]^ When contrasted with traditional static culturing of brain organoids, cultivating brain organoids in an environment with flowing nutrients results in a more robust and complex neural network.^[^ ^68^ ^]^ Utilizing microfluidic systems allows for the development of longer‐lasting and more biologically intricate brain‐on‐a‐chip systems.

#### 2.3.1 Microfluidics Enhances Nutrient and Metabolic Exchange in Brain Organoids

**Figure 5:**
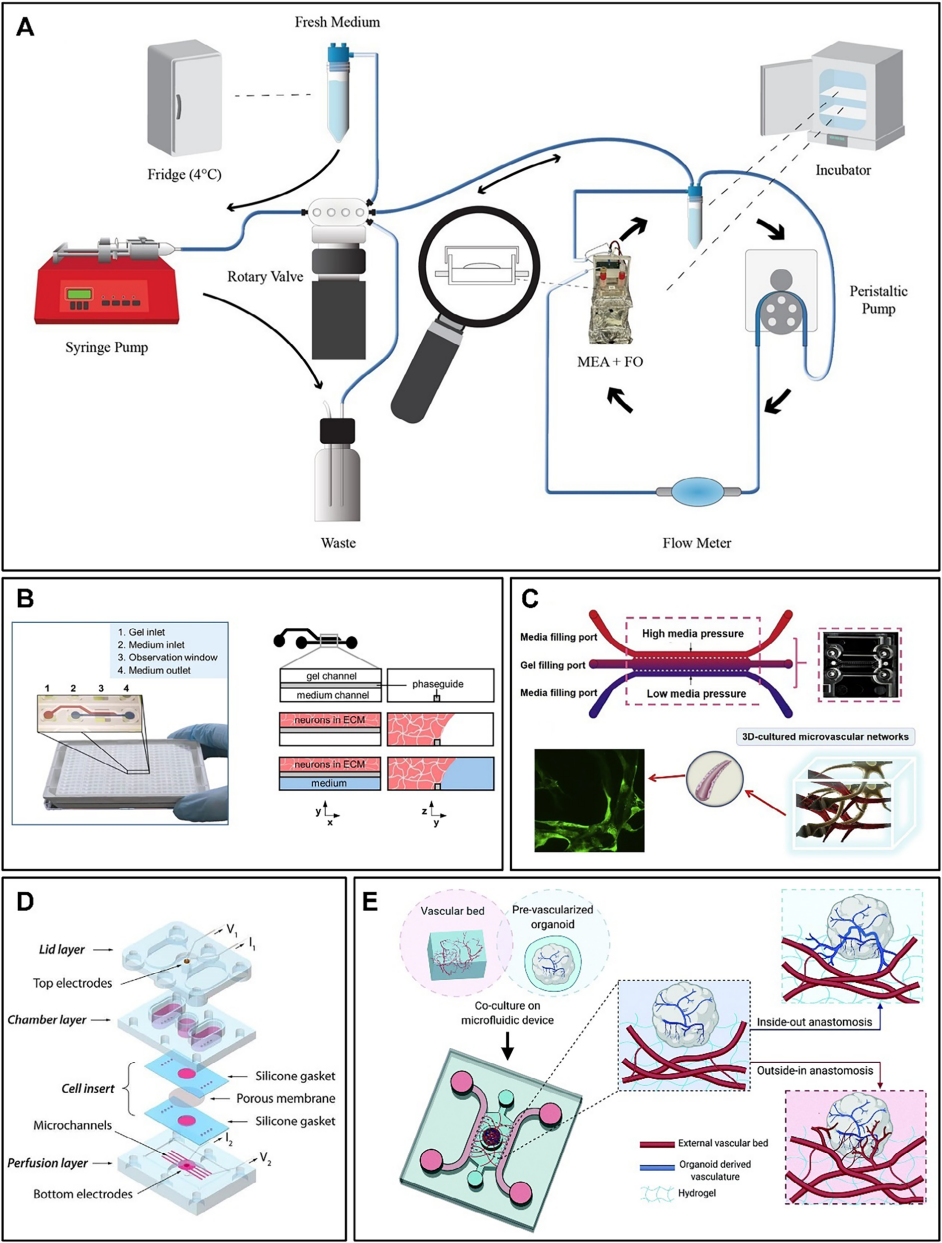
Microfluidic technology in brain‐on‐a‐chip. A) Neuroplatform's microfluidic system, including syringe pumps, rotary pumps, and peristaltic pump flowmeters.^[^ ^34^ ^]^ B) OrganoPlate physical image and flow diagram of the protocol for culturing a 3D neuron‐glial cell network.^[^ ^71^ ^]^ C) Schematic diagram of a human 3D microvascular microfluidic device and a schematic diagram of a 3D hybrid cell line cell gel after four days of culture.^[^ ^75^ ^]^ D) Schematic exploded view of the microfluidic platform for modeling the BBB.^[^ ^76^ ^]^ E) Schematic diagram of a possible mechanism for establishing a coincidence between vascular beds and pre‐vascularized organoids in vitro when organoids are cultured on microfluidic chips.^[^ ^67^ ^]^ (A) Reproduced under terms of the CC‐BY license.^[^ ^34^ ^]^ Copyright 2024, published by Frontiers. (B) Reproduced under terms of the CC‐BY license.^[^ ^71^ ^]^ Copyright 2016, published by Springer Nature. (C) Reproduced with permission.^[^ ^75^ ^]^ Copyright 2019, published by Elsevier. (D) Reproduced with permission.^[^ ^76^ ^]^ Copyright 2016, published by John Wiley and Sons. (E) Reproduced under terms of the CC‐BY license.^[^ ^67^ ^]^ Copyright 2021, published by Royal Society of Chemistry.

Microfluidic technology can precisely manipulate fluids in the micro‐nanoscale space. The interstitial fluid in the human brain plays a crucial role in transporting nutrients and removing metabolic wastes. Through microfluidic technology, it is possible to simulate the interstitial fluid and inject supplementary neuronal medium into the brain‐on‐a‐chip, thus maintaining the life of the organoids on the MEA.^[^ ^69^, ^70^ ^]^ To provide the medium to the organoids, a microfluidic system in brain‐on‐a‐chip needs to have syringe pumps, rotary valves, peristaltic pumps, and flow sensors^[^ ^34^ ^]^ (**Figure** **5**A). MEMS technology is required to realize microfluidic function on brain‐on‐a‐chip, which needs to provide growth space for cell/extracellular matrix mixture and channels for supplying growth medium^[^ ^71^ ^]^ (Figure 5B). The inlet and outlet of the medium can be utilized to refresh the medium and supply compounds and staining reagents to the cells. Microfluidic technology enables the damage‐free exchange of nutrients, gases, and waste products. This advancement allows for long‐term computational and decision‐making tasks in brain‐on‐a‐chip systems, which is crucial for maintaining the stability of brain organoids and facilitating Wetware Computing.

#### 2.3.2 Microfluidics Enables the Construction of Complex Brain Models

Microfluidics facilitates the creation of intricate brain models that simulate a more realistic human brain physiological environment.^[^ ^72^ ^]^ This includes replicating the blood‐brain barrier and the brain's vascular network. By constructing these complex brain models, we can achieve a representation that is closer to the actual physiological state. This advancement allows for a more comprehensive simulation of the complete brain, enhancing both the computational power and the long‐term stability of brain‐on‐a‐chip technologies.

In the body, the central nervous system consists of two main components: nerves and blood vessels. The nerve tissue is separated from the blood vessel chambers by the blood‐brain barrier (BBB). Brain‐on‐a‐chip can mimic multi‐layered tissue structures, and the molecular transport interface between cerebral blood vessels and histiocytes (e.g., astrocytes and pericytes) is a typical example of it.^[^ ^73^ ^]^ BBB can be simulated using a three‐layer polydimethylsiloxane (PDMS) chip. The upper and lower layers represent the blood vessel and nerve tissue chambers, respectively, and are separated by a porous PDMS membrane that acts as the BBB.^[^ ^11^ ^]^ The OrganoPlate 3D cell culture platform can be used to culture cells in quadruplicate and construct a 3D model of the BBB.^[^ ^74^ ^]^ Yang Wu et al.^[^ ^75^ ^]^ developed a microfluidic device featuring a central 3D fibrin hydrogel region containing GFP‐HBMEC and HA to simulate the BBB (Figure 5C). Ying I. Wang et al.^[^ ^76^ ^]^ modeled the blood‐brain barrier using a multi‐layer microfluidic chip structure, with the bottom layer containing microchannels and bottom electrodes, the middle layer forming neuronal chambers, the top layer preventing evaporation, and the cell insert assembled between the bottom and middle layers (Figure 5D).

In addition, microfluidics can also realize the construction of vascular networks on brain organoids.^[^ ^77^, ^78^, ^79^ ^]^ The vascular system is essential for transporting nutrients, oxygen, and metabolic waste products in biological tissues. Research has shown that brain organoids cultured in vitro are capable of developing vascular networks.^[^ ^80^ ^]^ By introducing a network of blood vessels, the lifespan and volume of brain organoids can be significantly improved.^[^ ^67^ ^]^ Current in vitro vascular models primarily fall into two distinct classes: self‐assembling and pre‐designed architectures^[^ ^67^ ^]^ (Figure 5E). In self‐organizing vascular networks, endothelial cells (ECs) are either incorporated into the hydrogel precursor solution prior to crosslinking or seeded onto the polymerized gel channel surfaces. Through gas exchange and nutrient transport with the mediator channel, the ECs in the hydrogel can self‐organize to form the microvascular network (MVN).^[^ ^78^ ^]^ In contrast, the pre‐patterned vascular network is created by first establishing a hydrogel stent, followed by the injection of ECs. This allows for the predetermined design of the vascular network's structure, direction, and geometry, facilitating the creation of more complex vascular networks.^[^ ^81^ ^]^

## 3 Data Encoding and Decoding Strategies for Brain‐on‐a‐Chip

In the Wetware Computing task, the data to be processed is transmitted to the brain‐on‐a‐chip through a certain method, and the calculation result is parsed according to the “response” of the brain‐on‐a‐chip, which corresponds to the encoding and decoding of the data.

### 3.1 Stimulation Signal Encoding for Brain Organoids

Multi‐modal stimulation signal delivery can be achieved using the brain‐on‐a‐chip platform. For example, chemical stimulation by adding chemical agents to the environment in which brain organoids grow^[^ ^82^ ^]^; temperature stimulation by changing the outside temperature^[^ ^83^ ^]^; ultrasound stimulation by applying ultrasound waves of different intensities^[^ ^84^ ^]^; magnetic stimulation by employing an applied magnetic field^[^ ^85^ ^]^; light stimulation by applying different wavelengths of light^[^ ^86^, ^87^ ^]^; electrical stimulation by direct application of electrical signals.^[^ ^88^ ^]^ Among them, electrical stimulation is the most commonly used in Wetware Computing tasks due to the easy adjustment of parameters and the relatively simple design of stimulation devices.

#### 3.1.1 Using Chemical Signals for Stimulation

Neurotransmitters are mediators that transmit signals between neurons, and chemicals can indirectly regulate neuronal excitability by modulating the release of neurotransmitters.^[^ ^89^ ^]^ The excitability of neurons determines the frequency and intensity at which they trigger action potentials, and therefore, changes to excitability directly affect the signaling and functional state of neural networks.^[^ ^82^ ^]^ Neurotransmitters are fundamentally categorized as either inhibitory, diminishing neuronal responsiveness, or excitatory, amplifying neural activity.^[^ ^90^ ^]^

**Figure 6:**
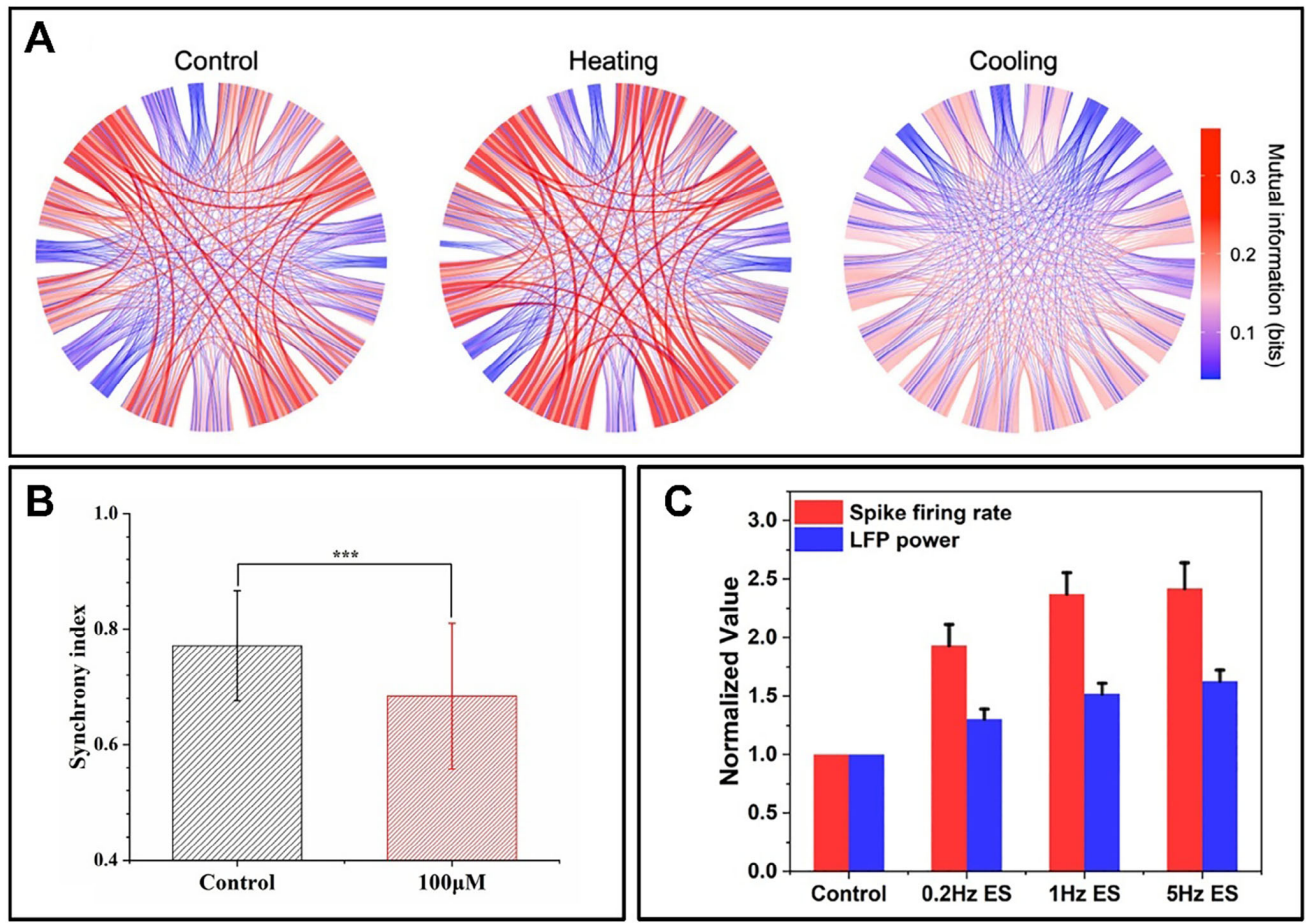
Stimulation and Response of in Vitro Organoids. A) Spatiotemporal dynamics graph of the Functional network based on control phase mutual information (MI), heating phases, and cooling phases.^[^ ^50^ ^]^ B) Mean synchronization index of neuronal firing before and after glycine treatment.^[^ ^90^ ^]^ C) Effect of different frequencies of electrical stimulation on hippocampal neuronal activity.^[^ ^38^ ^]^ (A) Reproduced with permission.^[^ ^50^ ^]^ Copyright 2024, published by American Chemical Society. (B) Reproduced with permission.^[^ ^90^ ^]^ Copyright 2024, published by Elsevier. (C) Reproduced with permission.^[^ ^38^ ^]^ Copyright 2022, published by American Chemical Society.

Inhibitory neurotransmitters play a vital role in neuron function by activating chloride or potassium channels, leading to hyperpolarization. This hyperpolarization contributes to their inhibitory effects. The most common inhibitory neurotransmitters are GABA (γ‐aminobutyric acid) and glycine, which primarily activate chloride channels. Activation of GABA_A receptors by GABA or GlyR by glycine triggers chloride ion influx through the opened channels, resulting in neuronal membrane hyperpolarization^[^ ^91^ ^]^ (**Figure** **6**B). The engagement of GABA with GABA_B receptors activates potassium channels and allows potassium ions to flow out of neurons, which also plays a role in hyperpolarizing neuronal membranes.^[^ ^92^ ^]^ Membrane hyperpolarization widens the differential between resting potential and action potential threshold, thereby elevating the depolarization energy requirement and reducing neuronal firing propensity.^[^ ^82^ ^]^

Excitatory neurotransmitters enhance neuronal excitability by binding to receptors in postsynaptic neurons, triggering depolarization. Glutamate serves as the major excitatory chemical messenger throughout the central nervous system. By binding to the AMPA receptor and the NMDA receptor, it can open the sodium and calcium channels, respectively, allowing sodium and calcium ions to enter the cell, causing depolarization.^[^ ^93^, ^94^ ^]^ Besides, acetylcholine activates sodium ion channels through nicotinic acetylcholine receptors, leading to depolarization and enhancing neuronal excitability.^[^ ^95^ ^]^

In brain‐on‐a‐chip systems, stimulus signals mainly play a role in encoding information, so it is necessary to use automated methods to achieve chemical stimulation. An ultraviolet light control uncaging system is used in Neuroplatform^[^ ^34^ ^]^ to realize chemical stimulation. Its principle is that a specific wavelength of light is used to break the molecular “cage” containing neuroactive molecules, thereby enabling the release of neurotransmitters. While chemical stimulation offers superior specificity and effectiveness in neural modulation, its implementation necessitates sophisticated instrumentation (**Table** **1**).

**Table 1:** Encoding Capabilities of Neural Stimulation Methods: Chemical, Thermal, and Electrical.

Stimulation	Temporal‐Spatial Precision	Effectiveness	Safety	Implementation Difficulty	Flexibility	Specificity
Chemical	Medium	High	High	Complex	Low	High
Thermal	Low	High	Low	Simple	Low	Low
Electrical	High	High	Low	Medium	High	Low

#### 3.1.2 Using Thermal Signals for Stimulation

Neuronal cells can sense the outside temperature and thus respond to stimuli of temperature signals.^[^ ^83^ ^]^ For individual neurons, increased temperature will increase the activation rate of sodium, potassium, and calcium channels, resulting in neurons being able to release action potentials at higher frequencies and enhance their excitability.^[^ ^96^ ^]^ Under low temperatures, ion channel kinetics slow, reducing neuronal excitability. This is compensated by increased network synchronization, maintaining information fidelity despite lower mutual information between neurons^[^ ^50^ ^]^ (Figure 6A).

While thermal signals can effectively stimulate brain organoids, the frequency and mode of stimulation are crucial for conducting Wetware Computing. The change in thermal signals occurs relatively slowly, making it challenging to encode data by adjusting temperature. As a result, the use of thermal signal stimulation in Wetware Computing tasks involving brain‐on‐a‐chip systems is quite rare (Table 1).

#### 3.1.3 Using Electrical Signals for Stimulation

Electrical stimulation signals can transmit different information by altering the amplitude, waveform, frequency, or spatiotemporal distribution of it.^[^ ^88^ ^]^ Electrical stimulation plays a vital role in neuronal networks, which have a profound impact on the regulation of neural networks by modifying signal transmission and the connectivity between synapses (Table 1).

Electrical stimulation signals can be divided into single‐phase electrical stimulation and biphasic electrical stimulation according to polarity, and they can also be divided into voltage stimulation and current stimulation according to the type of electrical stimulation.^[^ ^97^, ^98^ ^]^ Biphasic voltage stimulation that applies a negative pulse first, followed by a positive pulse, demonstrates higher efficiency in eliciting a network response, which is why it is widely utilized by researchers^[^ ^8^, ^38^ ^]^ (Figure 6C). The frequency of the electrical stimulation signal also has a significant influence on the emission of the spike signal, which affects the output of the neural network.^[^ ^99^ ^]^ Low‐frequency electrical signals are efficient for stimulating biological neural networks, enabling them to demonstrate learning capabilities.^[^ ^100^ ^]^ The specific parameters for effective electrical stimulation may vary due to differences in experimental equipment, conditions, cell types, and other factors. Typically, the amplitude of electrical stimulation is kept below 500 mV, while the frequency is generally selected within the range of 0.2 Hz to 500 Hz. This selection considers the effects of stimulation, safety, and long‐term stability. Furthermore, the electrical stimulation signal can encode input information through the spatiotemporal distribution of the stimulation. This means that both the timing intervals of the electrical stimulation and the specific application sites can be controlled.^[^ ^8^ ^]^ Pairwise training stimuli (PTS) can also induce plasticity changes in neural networks by applying stimulation to electrode pairs with a time delay.^[^ ^6^ ^]^

To encode information through electrical stimulation signals, researchers usually need to select several input electrodes on the MEA, define these electrodes as input regions, and then input electrical stimulation signals to brain organoids through these input region electrodes.^[^ ^7^ ^]^ In brain‐on‐a‐chip experiments, the frequency of the electrical stimulation signal can be encoded using various methods. Universal encoding techniques include linear encoding^[^ ^7^, ^9^ ^]^ and specific methods designed for particular scenarios.^[^ ^9^, ^63^ ^]^ For example, in obstacle avoidance tasks, information can be gathered employing a distance sensor, and the frequency of stimuli can be encoded linearly.^[^ ^7^, ^51^ ^]^ In the mechanical arm drawing task, the markers in the drawing area can be compared with the target geometry. When the drawing extends beyond the target area, different PTS combinations can be selected to encode the stimulus spatially.^[^ ^6^ ^]^ In the maze task, a specified burst stimulus can be delivered to the MEA when the robot either collides with an obstacle or when the target deviates by 90 degrees from the robot's direction of movement.^[^ ^63^ ^]^ In the ping‐pong game task, the electrical stimulation signal can be encoded using the position of the ball with a specific burst stimulus delivered to the MEA upon a successful shot or a miss.^[^ ^9^ ^]^

### 3.2 Electrophysiological Signal Decoding of Brain Organoids

The complexity of brain organoids generates large amounts of data during brain‐computer interactions. How to handle this data is a current topic of interest. Similar to the encoding of electrical stimulation signals, electrode regions need to be selected and predefined when decoding electrophysiological signals. It is feasible to specify the electrode region as the “output area” directly.^[^ ^9^ ^]^ However, it is better to ensure that the output area can respond to the stimulus promptly by first applying electrical stimulation to the stimulating electrode, then identifying the region that is more responsive to the stimulus and defining it as the output region.^[^ ^7^ ^]^ After the selection of electrodes is completed, it is necessary to remove the stimulus artifacts of the brain organoids and extract the action potentials of the brain organoids through the algorithms, to obtain the electrophysiological activities of the brain organoids. Brain organoids' electrophysiological activities can reveal their “instructions,” allowing for direct decoding. Additionally, machine learning algorithms can analyze the output characteristics of brain organoids for more accurate and efficient decoding.

#### 3.2.1 Removal of Stimulus Artifacts in Brain Organoids

Stimulus artifacts often occur during electrophysiological stimulation and electrical signal acquisition in the brain‐on‐a‐chip. Stimulus artifacts are captured and amplified by the diffusion of the stimulating current through tissues or electrolyte solutions inside and outside the organoids to the recording electrode, the presence of which can mask or interfere with the physiological electrical signals of the organism itself, thus affecting the accurate observation and analysis of the physiological activities of the organism.

Conventional methods for removing stimulus artifacts include interpolation, template subtraction, and model decomposition.^[^ ^101^ ^]^ Each method has trade‐offs between artifact removal efficacy and preservation of original signal details, requiring careful selection based on signal characteristics and experimental goals.

The interpolation‐based artifact removal algorithm operates on the principle of utilizing existing data information to estimate the lost or damaged portions of the data.^[^ ^102^ ^]^ This method has the advantage of being simple and easy to implement, but its heavy reliance on nearby data trends can excessively smooth genuine neural activity, leading to a loss of high‐frequency details. To mitigate this, adaptive windowing or combining interpolation with noise‐robust filters can help preserve finer signal features.^[^ ^103^ ^]^

The artifact removal algorithm based on template subtraction follows the general principle of constructing a typical template of artifacts and then subtracting this template from the recorded signal to achieve the effect of removing artifacts.^[^ ^103^ ^]^ This method is particularly well suited for dealing with electrical stimulation artifacts that are consistent in shape and timing, but imperfect template alignment or dynamic artifact variations may inadvertently remove overlapping neural signals.^[^ ^104^ ^]^ Iterative refinement of templates or hybrid approaches can improve balance.^[^ ^105^ ^]^

The artifact removal algorithm based on model decomposition adheres to the general principle of decomposing the signal into different components through mathematical modeling of the signal and artifact. The components are identified respectively, and the artifact is removed to retain the genuine neural activity signal.^[^ ^101^ ^]^ While powerful for complex artifacts, overfitting the model may distort genuine signals.^[^ ^104^ ^]^ Regularization techniques or validation against ground‐truth neural recordings can optimize the trade‐off.^[^ ^106^ ^]^

#### 3.2.2 Extraction of Action Potentials from Brain Organoids

The action potential is the main object that needs to be decoded by brain‐on‐a‐chip, and its relatively high spatial resolution and signal amplitude have high significance for the realization of decoding the “output instructions” of brain organoids. In the process of using action potentials to decode the “instructions” of brain organoids, the action potential needs to be separated from the original signal first.^[^ ^107^ ^]^

Traditional action potential extraction algorithms include the Gaussian Mixture Models method ^[^ ^108^ ^]^ Bayesian nonparametric method,^[^ ^109^ ^]^ fast peak Search method,^[^ ^110^ ^]^ etc. These separation algorithms have individual problems, such as the difficulty of separating overlapping spikes, the low computational efficiency in the calculation of large amounts of data, and the inability to evaluate the algorithms in the absence of genuine data. Some studies have separated overlapping spikes through iterative algorithms based on template matching or deconvolution, which significantly improves the quality of detection.^[^ ^111^, ^112^ ^]^ To address the issue of computational efficiency, some researchers use the “masking” vector to indicate whether a signal is detected on each electrode at each test, reducing the amount of data at the time of calculation.^[^ ^113^ ^]^ In addition, computational efficiency can also be optimized by merging firing data from the same neuron.^[^ ^114^ ^]^ Finally, in the absence of genuine data, it is also possible to evaluate the separation algorithms by using partial genuine data, using a designed model, or by its stability.^[^ ^109^, ^111^, ^112^, ^115^ ^]^

#### 3.2.3 Decoding Brain Organoid Data

After completing stimulus artifact recovery and action potential extraction from brain organoids, the next step is to decode the extracted data. This decoding process is necessary to interpret the signals generated by the brain organoids. The signals can be decoded directly by using the emission of action potentials, or using machine learning to decode brain organoids' signals.

**Figure 7:**
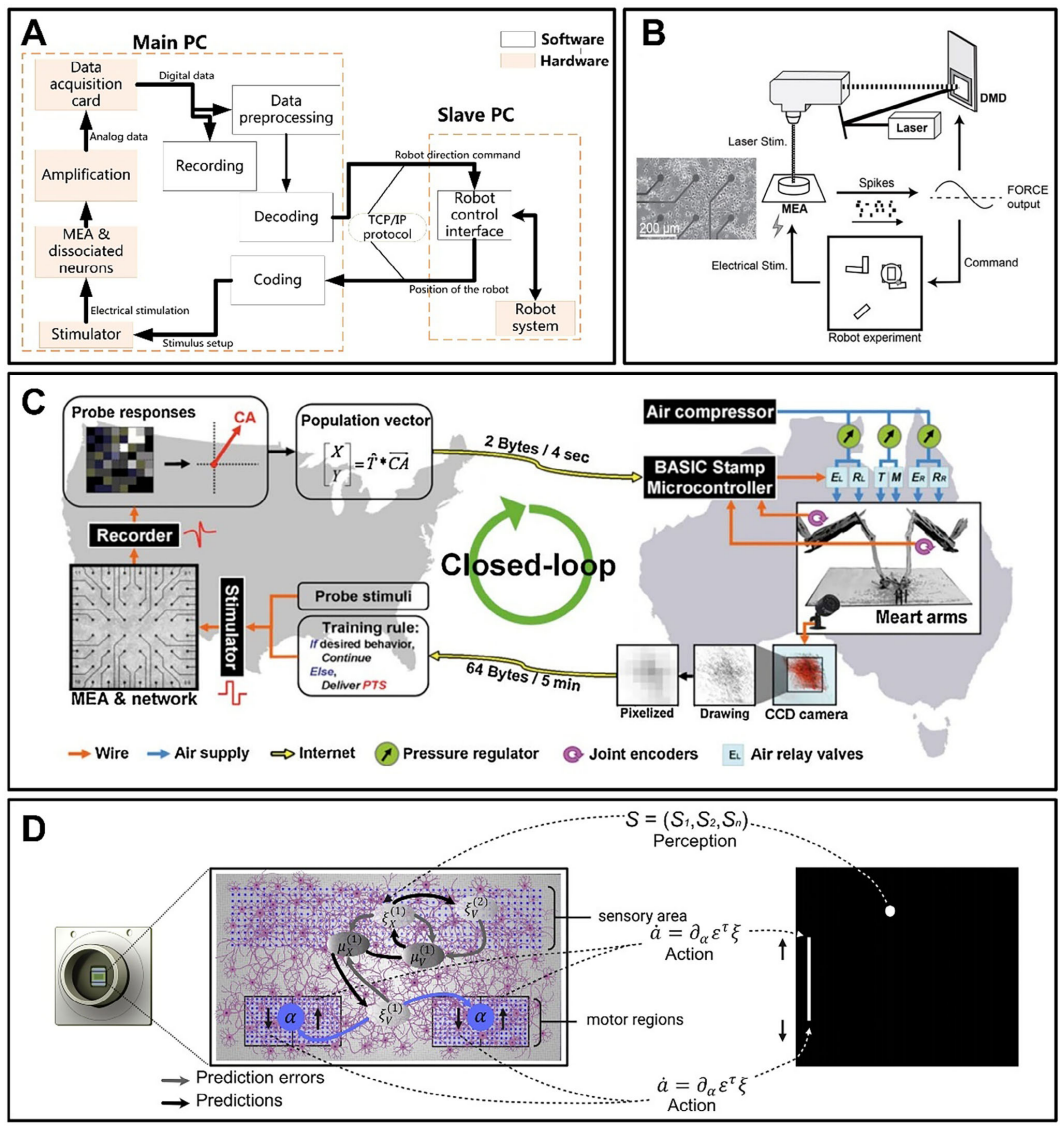
Encoding and decoding scheme of brain‐on‐a‐chip. A) The framework of the neuro‐robot system (Hierarchical dissociated neural network controls the robot to reach the objects).^[^ ^51^ ^]^ B) Schematic of the experimental system for physical reservoir computing (Using a living neuronal culture to perform pathfinding tasks).^[^ ^63^ ^]^ C) Schematic diagram of the MEART System (Isolated Neural Network controls robotic arm).^[^ ^6^ ^]^ D) Schematic diagram of the DishBrain system (In vitro neural network participates in the game world).^[^ ^9^ ^]^ (A) Reproduced under terms of the CC‐BY license.^[^ ^51^ ^]^ Copyright 2021, published by Public Library of Science. (B) Reproduced under terms of the CC‐BY license.^[^ ^63^ ^]^ Copyright 2021, published by AIP Publishing. (C) Reproduced with permission.^[^ ^6^ ^]^ Copyright 2007, published by Frontiers. (D) Reproduced under terms of the CC‐BY license.^[^ ^9^ ^]^ Copyright 2022, published by Elsevier.

Due to the “all‐or‐nothing” frequency coding mechanism of action potentials, decoding is typically achieved by counting the number of times a neuron emits an action potential over a specific period, known as the firing rate. In the task of controlling the robot's movement, the emission rate can be used to linearly map the wheel velocity and regulate the wheel of the robot, to realize the decoding of electrophysiological signals^[^ ^7^, ^51^ ^]^ (**Figure** **7**A). It is also possible to control the rotation speed of the robot's left and right wheels according to the error between the output signal and the target signal to realize the robot's control.^[^ ^63^ ^]^ In the robotic arm mapping task, the “Center of Activity” (CA) method, in which the position of the discharged neurons is weighted averaged over a specific time window, is used to determine the position of the “center” for decoding^[^ ^6^ ^]^ (Figure 7C). In the ping‐pong game task, the up‐and‐down movement of the “paddle” is controlled by the action potential emission rate of the two‐part region^[^ ^9^ ^]^ (Figure 7D). It is also possible to decode the output of the organoid by detecting synchronized bursts (a rapid sequence of spikes emitted by a neuron or a neural population within a short time window, followed by a period of silence^[^ ^116^ ^]^) across subnets and mapping them to predefined robotic actions, without requiring continuous rate‐velocity conversion.^[^ ^117^ ^]^

Applying machine learning to brain organoid signal decoding is an efficient way, on the one hand, it is helpful to optimize the growth environment of organoids in vitro and monitor them more accurately^[^ ^118^ ^]^; on the other hand, it can also carry out efficient and accurate clustering and regression prediction of the output of brain organoids for more direct decoding. The decoded results can be used not only for computational purposes but also as biofeedback to facilitate organoid learning.^[^ ^5^ ^]^ Machine learning algorithms commonly used for decoding electrophysiological data include Support Vector Machines(SVM), Convolutional Neural Network(CNN), Naive Bayesian Algorithm, K‐Nearest Neighbor Classification Algorithm(KNN), and Random Forest Algorithms.^[^ ^119^, ^120^, ^121^ ^]^ In addition to this, it is also possible to take pictures of brain organoids and then use machine learning to recognize the images to achieve cell analysis and output prediction.^[^ ^122^ ^]^ In Reservoir Computing, which has been popular recently, biochips have been proven to achieve the function of hardware reservoirs. FORCE learning is an adaptive algorithm that adjusts the weights of a neural network to minimize the error between the output signal and the target signal. This method is particularly useful in reservoir computing because it allows the network to produce coherent output from its spontaneous activity without the need for complex adjustments to the network's internal dynamics. FORCE learning can generate coherent signal outputs from a living neuronal culture, and these output signals are used as feedback signals to control a mobile vehicle robot in a maze^[^ ^63^ ^]^ (Figure 7B). By introducing the linear regression function as the decoding function of neural activity, their electrophysiological interactions can be linearly read out, which can achieve various complex tasks.^[^ ^8^ ^]^

## 4 Wetware Computing Applications for Brain‐on‐a‐Chip

The application of brain‐on‐a‐chip technology in Wetware Computing has been significantly enhanced by recent advancements in in vitro brain‐computer interface technology. On one hand, the microsystem research platforms designed for brain‐on‐a‐chip applications are continually being improved. On the other hand, these brain‐on‐a‐chip microsystems demonstrate high‐performance Wetware Computing capabilities and exhibit remarkable learning potential.

### 4.1 Brain‐on‐a‐Chip Microsystem Research Platform

The brain‐on‐a‐chip microsystem research platform usually includes a brain organoid, a microelectrode array, a microfluidic system, a stimulus signal output system, data processing system.^[^ ^123^ ^]^ As a research platform for Wetware Computing applications, it plays an important role in supplying energy to brain organoids, generating stimulation signals, and encoding and decoding data.^[^ ^5^ ^]^

Several companies have developed commercial brain‐on‐a‐chip research platforms at present. FinalSpark, a leading Swiss biocomputing company, has launched its first online platform, Neuroplatform,^[^ ^34^ ^]^ enabling researchers around the world to access 16 human brain organoids around the clock. Neuroplatform's hardware architecture includes microelectrode arrays, electrophysiological stimulation and recording systems, microfluidic systems, photographic systems for recording the state of organoids, Chemical stimulation systems, and environmental monitoring systems. It can perform stable and efficient multimodal stimulation and action potential monitoring of organoids. Dutch company Mimetas has developed a technology platform called OrganoPlate,^[^ ^71^ ^]^ which is a microfluidic 3D tissue culture plate that supports 96 tissue models in one plate. By utilizing a patented liquid processing technology called PhaseGuide, the OrganoPlate enables free interaction and migration of cells between channels, as well as membrane‐free co‐culture of extracellular matrices, optimized microenvironments, and perfused tubular tissues. In addition, continuous media perfusion via the microfluidic network in the OrganoPlate can mimic blood flow and enable the exchange of nutrients, oxygen, and metabolites.

### 4.2 Wetware Computing Performance of Brain‐on‐a‐Chip

Brain‐on‐a‐chip has demonstrated its feasibility in Wetware Computing tasks across various applications. Furthermore, the brain‐on‐a‐chip has exhibited remarkable potential in Wetware Computing, particularly in terms of learning efficiency and required training epochs (**Table** **2**).

**Table 2:** Summary of Wetware Computing Tasks for brain‐on‐a‐chip and Their Implementations.

Wetware Computing Tasks	Implementation	Decoding Scheme	Encoding Scheme	Evaluation Parameters	Evaluation Result	Refs.
Speech Recognition	3D Organoid	Firing Rate + Logistic Regression	Electrical Stimulation	Accuracy	78.0% ± 5.2%	H. Cai et al. ^[^ ^8^ ^]^
Predicting Nonlinear Chaotic Equations	3D Organoid	Firing Rate + Linear Regression	Electrical Stimulation	Regression Score	0.8233	
Pathfinding	2D Neural Network	Firing Rate + Regional Selective Decoding	Electrical Stimulation	Time To Reach Object / Correct Turning Percentage	279 s/≈100%	Yongcheng Li et al.^[^ ^49^ ^]^
	2D Neural Network	Firing Rate + FORCE Learning	Electrical + Light Stimulation	Path Completion Status	Complete	Yuichiro Yada et al. ^[^ ^62^ ^]^
Playing Video Games	2D Neural Network	Firing Rate + Regional Selective Decoding	Electrical Stimulation	Average Rally Length / Aces / Long Rallies	Increase / Decrease / Increase	Brett J. Kagan et al.^[^ ^9^ ^]^
Controlling Robotic Arm	2D Neural Network	Firing Rate + Center of Activity	Electrical Stimulation	Painting Results	——	Douglas J. Bakkum et al.^[^ ^6^ ^]^
Obstacle Avoidance	2D Neural Network	Firing Rate + Linear Decoding	Electrical Stimulation	Average Distance Between Hits	≈70 Pixels	Jacopo Tessadorit et al.^[^ ^7^ ^]^
	Spiking Neural Networks (Algorithm)	Bursts + Spike‐Timing‐Dependent Plasticity	Electrical Stimulation	Learning Quality (Q) / Number of Collisions	≈0.8 / Decrease Exponentially with The Increase In Q	Sergey A. Lobov et al.^[^ ^113^ ^]^

**Figure 8:**
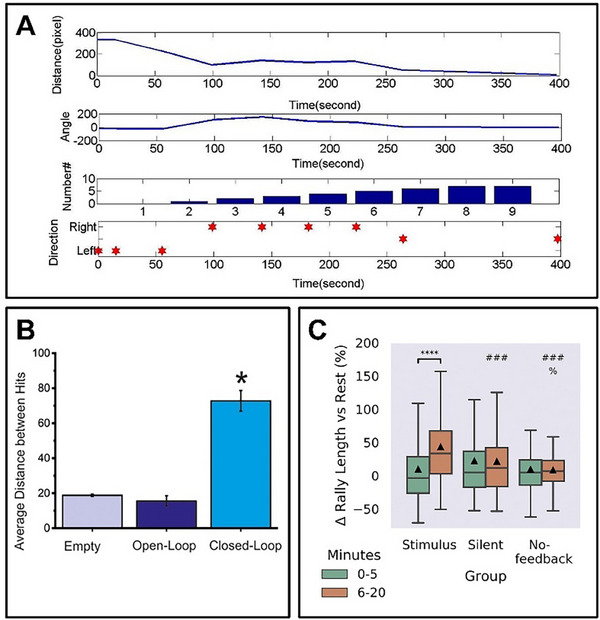
Brain‐on‐a‐chip Wetware Computing performance. A) The performance of the neural network in the pathfinding task, showing the distance between the object and the robot, the relative position between the object and the robot, the correct time for the robot to execute the command, and the direction determined by the neural network.^[^ ^51^ ^]^ B) Performance of robot controlled by the neural network in obstacle avoidance tasks (average distance between two consecutive collisions, calculated in pixels). Closed‐loop experiments have the best results.^[^ ^7^ ^]^ C) In the game world, neural networks are stimulated using different feedback schemes, and they show significant learning ability and longer rally length compared with silence and no feedback conditions.^[^ ^9^ ^]^ (A) Reproduced under terms of the CC‐BY license.^[^ ^51^ ^]^ Copyright 2021, published by Public Library of Science. (B) Reproduced under terms of the CC‐BY license.^[^ ^7^ ^]^ Copyright 2021, published by Frontiers. (C) Reproduced under terms of the CC‐BY license.^[^ ^9^ ^]^ Copyright 2022, published by Elsevier.

In the pathfinding task, the time it takes to find the target decreases as the number of training sessions increases, from 360 s to 316 s, to 279 s, and the correct turning percentage during this period are 67%, 75%, and 100%, respectively^[^ ^51^ ^]^ (**Figure** **8**A). it is also possible that use brain‐on‐a‐chip to conduct a pathfinding task in a more complicated situation.^[^ ^63^ ^]^

In the obstacle avoidance task, the average travel distance between two impacts with closed‐loop feedback brain‐on‐a‐chip is significantly higher than that of the empty chip and the open‐loop controlled brain‐on‐a‐chip, which further proves that the brain‐on‐a‐chip can learn from external stimuli and perform effective control^[^ ^7^ ^]^ (Figure 8B).

In the speech recognition task, the accuracy achieved by the brain‐on‐a‐chip was 78.0% ± 5.2%.^[^ ^8^ ^]^ This prediction accuracy is significantly higher than that of the logistic regression algorithm and comparable to that of an artificial neural network (ANN). However, the brain‐on‐a‐chip requires fewer data points, demonstrating its superior learning efficiency.^[^ ^8^ ^]^

In the task of predicting nonlinear chaotic equations, brain‐on‐a‐chip can save 90% of the training time compared with the artificial neural network using LSTM and can achieve similar prediction results, which proves the great advantage of brain‐on‐a‐chip over silicon‐based chips in training time (**Table** **3**).

**Table 3:** Performance comparison of Brain‐on‐a‐Chip with artificial intelligence algorithm in nonlinear chaotic equation prediction.

	Brain‐on‐a‐Chip [Epoch = 4]	Linear Regression	ANN w/o LSTM [Epoch = 50]	ANN w LSTM [Epoch = 50]	ESN	Reference
Epoch	4	——	50	50	——	H. Cai et al. ^[^ ^8^ ^]^
Regression Score	0.8233	0.1283	0.5442	0.8934	0.9397	

Besides, brain‐on‐a‐chip has demonstrated extremely high accuracy and computational performance in tasks such as controlling a robotic arm drawing^[^ ^6^ ^]^ and playing video games^[^ ^9^ ^]^ (Figure 8C). These results highlight the learning capabilities of the brain‐on‐a‐chip and its viability for computational tasks and decision‐making. Recent advances in SNN‐based modeling successfully emulate key bio‐neural mechanisms, demonstrating exceptional performance with zero‐collision obstacle avoidance while providing a scalable platform for future brain‐on‐a‐chip implementations.^[^ ^117^ ^]^

### 4.3 The Main Technical Challenges of Brain‐on‐a‐Chip in Wetware Computing Applications

Despite having demonstrated strong learning capabilities in Wetware Computing applications and thereby proving the feasibility of its application in this field, brain‐on‐a‐chip still faces several technical issues that need to be resolved.

First, cultivating more complex brain organoids is one of the challenges.^[^ ^12^ ^]^ Although brain organoids already possess more complex structures and functions compared to biological neural networks, they still have significant gaps compared to real brains in terms of cell types and gene expression, complex regionalization and neural circuitry, developmental stages, and maturity.^[^ ^23^ ^]^ These differences affect the accuracy of their simulation of learning and memory processes and limit the functions of brain organoids.

In addition, designing microfluidic systems that can support large‐scale brain organoids is another urgent challenge to be addressed.^[^ ^67^ ^]^ At present, most brain organoids in Wetware Computing applications are avascular and rely on passive diffusion to supply nutrients and remove waste.^[^ ^68^ ^]^ This restricts the size, complexity, and stability of brain organoids.

Moreover, there are many technical difficulties in electrically stimulating and recording brain organoids as well as processing the data. These include developing 3D microelectrode arrays that can record and stimulate brain organoids at higher resolution,^[^ ^44^, ^46^ ^]^ creating algorithms and tools that can process and analyze the large amount of electrophysiological signals generated by brain organoids,^[^ ^59^ ^]^ designing methods for encoding external data through multimodal stimulation signals,^[^ ^5^ ^]^ and devising more rational strategies for decoding electrophysiological signals.^[^ ^5^ ^]^

Lastly, as a cutting‐edge technology, reducing costs is also a very important challenge. The current technologies and equipment involved in brain‐on‐a‐chip are relatively expensive, which may limit their widespread application. Only by achieving standardized and automated system design can these costs be reduced, making it more viable.

## 5 Conclusion and Outlook

The application of brain‐on‐a‐chip to Wetware Computing is still a relatively cutting‐edge research direction, and there are relatively few articles on the biocomputing capabilities of 3D brain organoids. However, some studies have proven that biochips have strong performance and broad prospects in biocomputing. As the most complex organ in living organisms, the brain has processing power that is difficult to achieve on silicon‐based chips. Brain‐on‐a‐chip has the complexity and diversity necessary to mimic the human brain, as well as a high degree of plasticity and adaptability applied to the field of computing.

While organoid‐based computation and data processing face several challenges—such as maintaining long‐term organoid viability, complex neural encoding/decoding, intricate device integration, and large‐scale data management—current brain‐on‐a‐chip systems still lag behind conventional silicon‐based hardware and AI algorithms in computational efficiency and experimental reliability. Nevertheless, the technology's unique advantages, including energy efficiency, adaptive learning capacity, and rapid information processing, have garnered significant recognition.^[^ ^5^, ^8^, ^9^, ^51^, ^63^ ^]^ Advances in biotechnology and sensing platforms continue to expand the potential of Wetware Computing, positioning brain‐on‐a‐chip systems as a promising paradigm for future computational architectures.^[^ ^34^, ^40^, ^54^, ^55^, ^56^, ^71^ ^]^

In addition to serving as a biological processor to complete a series of computational and learning tasks, brain‐on‐a‐chip can also be used in pharmacology and pathology research, especially for solving neurodegenerative diseases.^[^ ^124^, ^125^ ^]^ Brain‐on‐a‐chip technology offers a platform for researchers to study human brain organs in vitro. This approach enables scientists to conduct fundamental research on cognitive mechanisms, brain tissue structure, and cell function. With the ongoing advancements in bioelectronics, brain‐on‐a‐chip is expected to play a significant role in various fields related to human brain research.

## Conflict of Interest

The authors declare no conflict of interest.

## Author Contributions

P.W., X.C., and J.L. played a crucial role throughout the entire process of the manuscript, from its initial conception and organization to the final revision. Their contributions have been instrumental in shaping the content, addressing the reviewers' concerns, and enhancing the overall quality of the paper. J.L. has made substantial contributions to this review article. Her expertise and insights have been invaluable throughout the entire process, from conceptualizing the review's scope to providing critical feedback during the writing and revision stages. Her involvement has been crucial in shaping the direction and depth of the review.
